# Acromegaly with congenital generalized lipodystrophy – two rare insulin resistance conditions in one patient: a case report

**DOI:** 10.1186/s13256-020-2352-9

**Published:** 2020-02-21

**Authors:** Vanessa Guerreiro, Irene Bernardes, Josué Pereira, Roberto Pestana Silva, Susana Fernandes, Davide Carvalho, Paula Freitas

**Affiliations:** 1 Department of Endocrinology, Diabetes and Metabolism, Centro Hospitalar Universitário de São João EPE, Alameda Professor Hernâni Monteiro, 4202-451 Porto, Portugal; 2 Faculty of Medicine of the Universidade do Porto, Porto, Portugal; 3 Instituto de Investigação e Inovação em Saúde, Universidade do Porto, Porto, Portugal; 4 Department of Neuroradiology, Centro Hospitalar Universitário de São João, Porto, Portugal; 5 Department of Neurosurgery, Centro Hospitalar Universitário de São João, Porto, Portugal; 6 Department of Pathology, Centro Hospitalar Universitário de São João, Porto, Portugal; 7 Department of Genetics, Faculty of Medicine, Universidade do Porto, Porto, Portugal

**Keywords:** Lipodystrophies, Acromegaly, IGF-1

## Abstract

**Background:**

Lipodystrophies are a group of diseases which are characterized by abnormal adipose tissue deposition and are frequently associated with metabolic changes. Congenital generalized lipodystrophy is an autosomal recessive syndrome, with a prevalence < 1:10 million. Acromegaly is a rare disease, secondary to the chronic hypersecretion of growth hormone and insulin-like growth factor-1, with characteristic metabolic and somatic effects. “Acromegaloidism” is a term used for patients who manifest clinical features of acromegaly, but do not present a demonstrable hormone growth hypersecretion. The extreme shortage of subcutaneous adipose tissues and muscle hypertrophy confer an acromegaloid-like appearance in these patients.

**Case presentation:**

We describe a case of a patient with the rare combination of Berardinelli–Seip congenital lipodystrophy and acromegaly; our patient is a 63-year-old white man, who was referred to an endocrinology consultation for suspected lipodystrophy. He had lipoatrophy of upper and lower limbs, trunk, and buttocks, with muscular prominence, acromegaloid facial appearance, large extremities, and soft tissue tumescence. In addition, he had dyslipidemia and prediabetes. His fat mass ratio (% trunk fat mass/% lower limbs fat mass) was 1.02 by densitometry and he also had hepatomegaly, with mild steatosis (from an abdominal ultrasound), and left ventricular hypertrophy (from an electrocardiogram). His first oral glucose tolerance test had growth hormone nadir of 0.92 ng/mL, and the second test, 10 months afterwards, registered growth hormone nadir of 0.64 ng/mL (growth hormone nadir < 0.3 ng/mL excludes acromegaly). Pituitary magnetic resonance imaging identified an area of hypocaptation of contrast product in relation to a pituitary adenoma and he was subsequently submitted to transsphenoidal surgical resection of the mass. A pathological evaluation showed pituitary adenoma with extensive expression of growth hormone and adrenocorticotropic hormone, as well as a rare expression of follicle-stimulating hormone and prolactin.

A genetic study revealed an exon 3/exon 4 deletion of the *AGPAT2* gene in homozygosity.

**Conclusions:**

Congenital generalized lipodystrophy is a rare disease which occurs with acromegaloid features. As far as we know, we have described the first case of genetic lipodystrophy associated with true acromegaly.

Although this is a rare association, the presence of congenital generalized lipodystrophy should not exclude the possibility of simultaneous acromegaly.

## Background

Lipodystrophies are a group of genetic or acquired diseases characterized by abnormal adipose tissue deposition, including peripheral fat loss (lipoatrophy) and central fat accumulation (lipohypertrophy or abdominal prominence), which could be present separately or combined in the same individual [1–3]. These syndromes are usually, but not invariably, linked with severe metabolic complications, such as insulin resistance, diabetes, lipid abnormalities, hypertension, and hepatic steatosis [4]. Many complications of lipodystrophy are secondary to deficient adipose mass, resulting in ectopic lipid storage in the liver, muscles, and other organs, with associated insulin resistance [5].

Furthermore, the extremely low levels of leptin, resulting from subcutaneous adipose tissue loss, play an important role in the pathophysiology of the associated comorbilities [6].

Congenital generalized lipodystrophy (CGL) or Berardinelli–Seip congenital lipodystrophy (BSCL) is a well-defined syndrome which is characterized by a generalized absence of adipose tissue from birth or shortly after, as well as severe insulin resistance [7, 8]. The condition is inherited as an autosomal recessive trait, which is often associated with parental consanguinity [9]. Several causative genetic mutations have been identified. There are at least four molecularly distinct forms where the variations of 1-acylglycerol-3-phosphate O-acyltransferase 2 (*AGPAT2*; BSCL type 1) and *BSCL2* (BSCL type 2) are the most common variations [10]. However, in most cases, the diagnosis of lipodystrophy is based on family history, physical examination, body composition, and metabolic status, supplemented by confirmatory genetic testing, albeit only in certain forms [5, 8]. There are no defined serum leptin levels that establish or rule out the diagnosis of lipodystrophy.

The AGPAT2 protein belongs to the family of AGPATs. These enzymes are fundamental for the biosynthesis of glycerol-3-phosphate triglycerides and phospholipids. The protein is highly expressed in adipose tissue, and its deficiency can cause lipodystrophy by inhibiting/reducing the synthesis of triacylglycerols and its storage in adipocytes. It is likely that a low activity of AGPAT2 could also increase the tissue levels of lysophosphatidic acid, which, in turn, could affect the functions of adipocytes [3, 7, 11].

Ever since Berardinelli described a very rare case of generalized congenital lipodystrophy in a 2-year-old boy from Brazil in 1954 [12], nearly 400 cases [13] have been reported in the literature, with a prevalence < 1 in 10 million [14].

With a prevalence of 38 to 80 cases per million, acromegaly is a rare disease. It is an insidious disease, which is secondary to the chronic hypersecretion of growth hormone (GH) and insulin-like growth factor-1 (IGF-1), with its characteristic metabolic and somatic effects. Most cases of acromegaly are caused by pituitary adenoma and the first-choice treatment of these cases is transsphenoidal surgery [15].

The extreme shortage of subcutaneous adipose tissue and other adipose tissues, and muscle hypertrophy, confer an acromegaloid-like appearance in patients with CGL [13]; however, no case of BSCL and true acromegaly has been reported in the literature to date.

## Case presentation

**Fig. 1:**
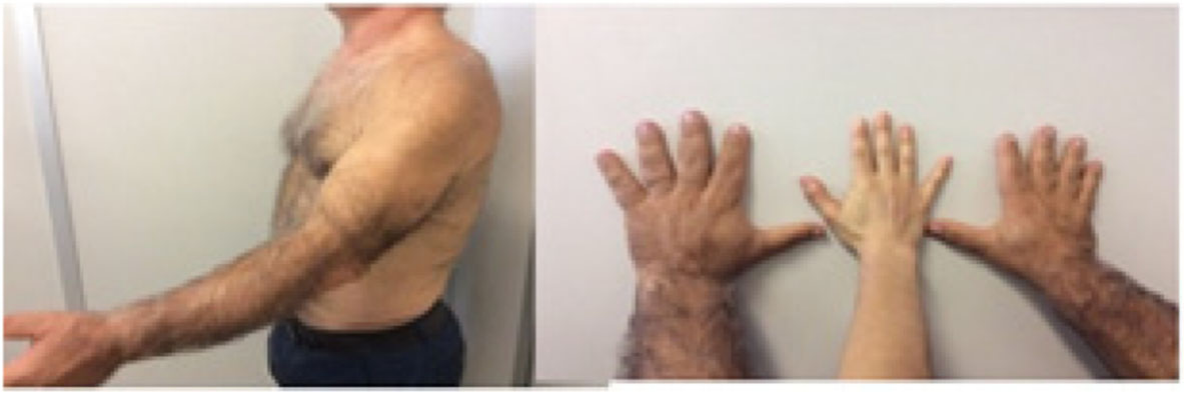
Picture of the patient

A 63-year-old white man (Fig. 1), who was born from a consanguineous union (his parents were first cousins), was referred to our department for suspected lipodystrophy.

He experienced uncomplicated prenatal and postnatal periods, with normal developmental milestones.

His 59-year-old sister has a similar phenotype, and had generalized lipoatrophy since childhood, without diabetes or hypertension, and with normal IGF-1. A physical examination revealed that neither of the parents had lipodystrophic changes and neither had diabetes.

The results of our patient’s physical examination were: weight 95.4 kg; height 1.72 m; body mass index (BMI) of 32.24 kg/m^2^; waist circumference 91 cm; blood pressure 119/75 mmHg; and near-total absence of subcutaneous fat on his upper and lower limbs, trunk, and buttocks, which is suggestive of generalized lipoatrophy. His facial appearance demonstrated a notable acromegaloid appearance, with thick lips, widening of the wings of the nose, creased nasolabial grooves, dental diastema, and prominence of the supra-ciliary arches, with slight prognathism. He also had muscular prominence, large hands and feet, and soft tissue tumescence, without acanthosis nigricans.

Biochemical tests (Table 1) revealed glycated hemoglobin (HbA1c) of 6.0%; insulin resistance – homeostatic model assessment of insulin resistance (HOMA-IR) 9.6; total cholesterol 187 mg/dL (normal range < 200 mg/dL); high-density lipoprotein (HDL) cholesterol 28 mg/dL (normal range > 60 mg/dL); low-density lipoprotein (LDL) cholesterol 96 mg/dL (normal range < 100 mg/dL); triglycerides 314 mg/dL (normal range < 150 mg/dL); macroalbuminuria in spot urine with 382.5 mg/g creatinine (normal range < 30 mg/dL); central hypothyroidism (CH) with free thyroxine (FT4) 0.65 ng/dL (normal range 0.70–1.48 ng/dL); and thyroid-stimulating hormone (TSH) 1.10 μUI/mL (normal range 0.35–4.94 μUI/mL). There were increased IGF-1 values of 379–481 ng/mL (normal range < 269 mg/dL). In the first oral glucose tolerance test (OGTT) (Table 2), our patient had impaired glucose tolerance (101 mg/dL glucose at 0 minutes, and 186 mg/dL at 2 hours), with GH at 0 minutes of 1.5 ng/mL and 0.92 ng/mL nadir (as we used an ultrasensitive assay – IMMULITE 2000 hGH – we could use a cut-off of a nadir serum < 0.3 ng/mL to exclude acromegaly [16]). As there was a high clinical suspicion of true acromegaly, we performed a second OGTT 10 months later (Table 3), which supported the diagnosis of diabetes (plasma glucose at 0 minutes of 120 mg/dL, and 204 mg/dL at 2 hours), and confirmed the diagnosis of acromegaly (GH of 0.98 ng/mL at 0 minutes, and 0.64 ng/mL nadir).

**Table 1:** Biochemical and hormonal parameters at diagnosis of acromegaly

Parameter	Result	Normal range
Fasting plasma glucose, mg/dL	101	75–110
Total cholesterol, mg/dL	187	< 200
Triglycerides, mg/dL	314	< 150
HDL, mg/dL	28	> 60
Calculated LDL, mg/dL	141	< 130
Urea, mg/dL	51	10–50
Creatinine, mg/dL	0.87	0.67–1.17
AST, U/L	38	10–37
ALT, U/L	36	10–37
GGT, U/L	46	10–49
IGF-1, nmol/L	379	94–269
FT4, ng/dL	0.65	0.70–1.48
TSH, μUI/mL	1.10	0.35–4.94
FSH, mUI/mL	3.98	1.50–12.4
LH, mUI/mL	5.48	1.70–8.60
Total testosterone, ng/mL	3.64	2.80.8.0
SHBG, nmol/L	34.3	14.5–48.4
Estradiol, pg/mL	25.9	7.6–42.6
Prolactin, ng/mL	7.7	4.0–15.2
Cortisol (a.m.), μg/dL	8.7	6.2–19.4
ACTH (a.m.), ng/L	26.9	< 63.3
HbA1c, %	6.0	4.0–6.0
Albuminuria, mg/L	314.0	< 30.0

*ACTH* adrenocorticotropic hormone, *ALT* alanine transaminase, *AST* aspartate transaminase, *FSH* follicle-stimulating hormone, *FT4* free thyroxine, *GGT* gamma-glutamyltransferase, *HbA1c* glycated hemoglobin, *HDL* high-density lipoprotein, *IGF-1* insulin-like growth factor-1, *LDL* low-density lipoprotein, *LH* luteinizing hormone, *SHBG* sex hormone-binding globulin, *TSH* thyroid-stimulating hormone

**Table 2:** First oral glucose tolerance test

OGTT (1st)	Glucose (mg/dL)	Insulin (μU/mL)	GH (ng/mL)
0 minutes	101	38.4	1.50
30 minutes	196	155.1	*0.98*
60 minutes	229	228.8	*0.93*
90 minutes	221	233.5	*0.92*
120 minutes	186	184.2	1.45

*GH* growth hormone, *OGTT* oral glucose tolerance test

**Table 3:** Second oral glucose tolerance test, 10 months later

OGTT (2nd, 10 months)	Glucose (mg/dL)	Insulin (μU/mL)	GH (ng/mL)
0 minutes	120	40.3	0.98
30 minutes	239	176.8	0.69
60 minutes	290	138.9	0.73
90 minutes	266	231.9	0.65
120 minutes	204	102.3	0.64

*GH* growth hormone, *OGTT* oral glucose tolerance test

Renal function, hepatic function, gonadal function, prolactin (PRL), morning adrenocorticotropic hormone (ACTH), and cortisol were all normal.

The fat mass ratio (FMR) by bone densitometry (% trunk fat mass/% lower limbs fat mass) was 1.02 (FMR in men ≥ 1.961 discriminates lipodystrophy [16]), without signs of osteoporosis (lumbar spine T score + 4.8, Z score + 4.6; femur T score + 4.2, Z score + 4.7).

Total colonoscopy and upper digestive endoscopy were normal. Hepatosplenomegaly (17 cm) with mild fatty liver was observed in an abdominal ultrasound. In an electrocardiogram, left ventricular hypertrophy was observed; mild concentric left ventricular hypertrophy with mean ventricular ejection fraction of 67% was observed in an echocardiogram.

**Fig. 2:**
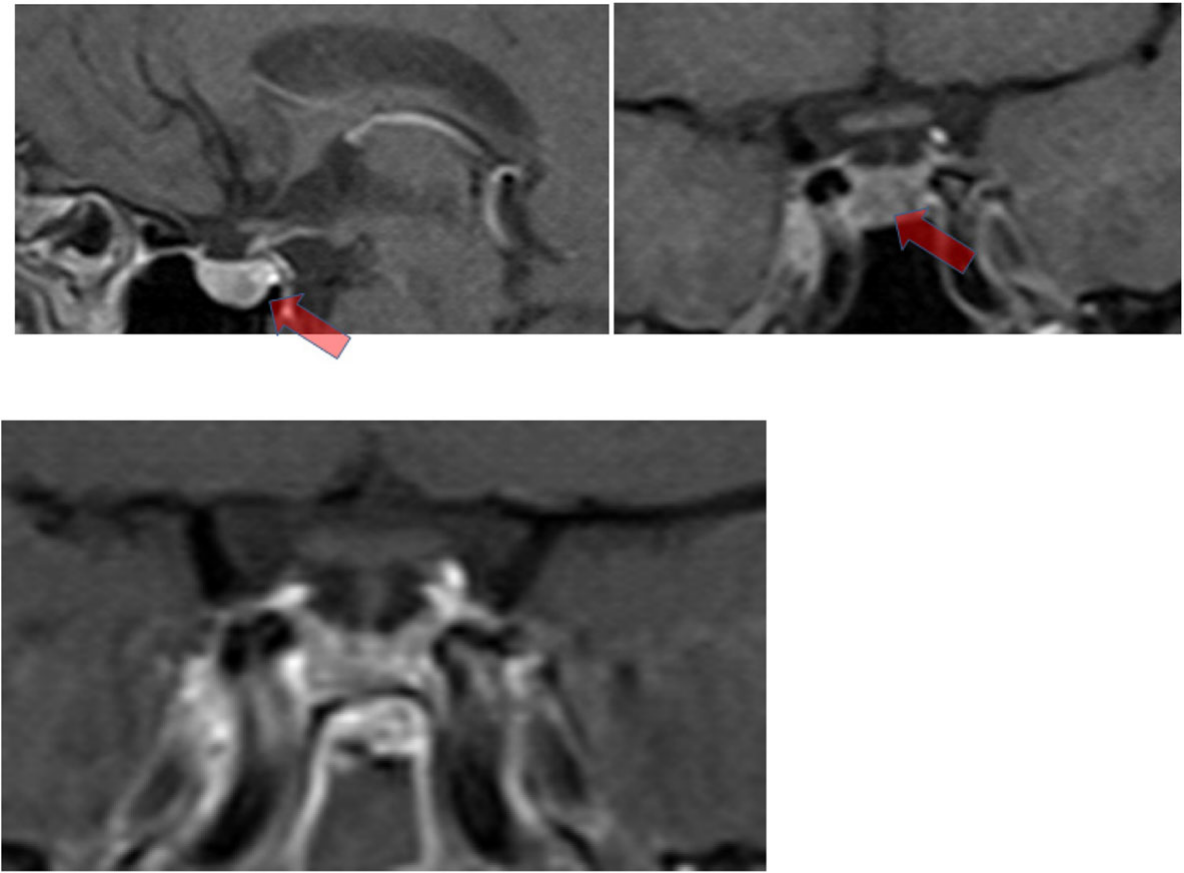
Pituitary magnetic resonance imaging. Arrow: The most hypocaptizing (darkest gray) region, with rounded aspect, in the right half of the pituitary gland, passing the midline to the opposite side and prophesying to the sphenoid sinus in relation to the pituitary adenoma

**Fig. 3:**
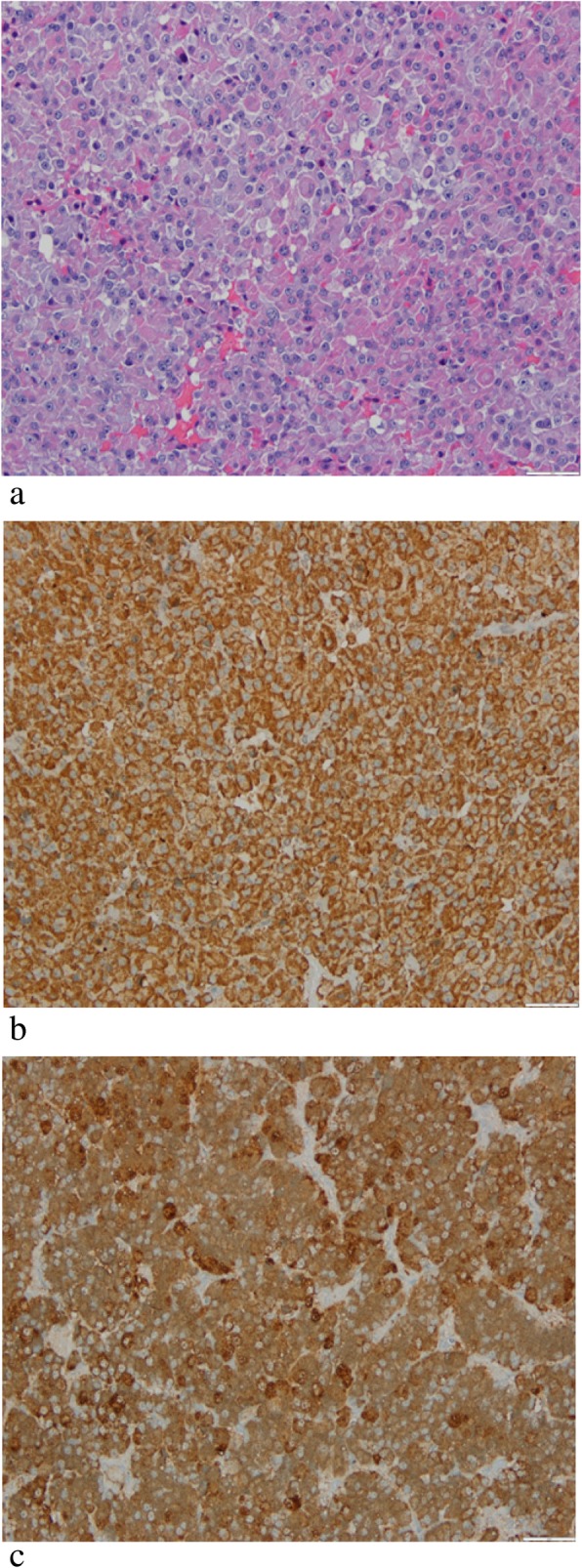
Histological examination. Pituitary adenoma with areas of cells with eosinophilic or amphiphilic cytoplasm (**a**) with somatotropin expression (**b**), and in other areas, cells with broad amphiphilic and granular cytoplasm with adrenocorticotropic hormone expression (**c**)

In the pituitary magnetic resonance imaging (MRI; Fig. 2), an area of hypocaptation of contrast product with rounded aspect in the right half of the pituitary gland was observed, passing the midline to the opposite side and invading the sphenoid sinus, in relation to the pituitary adenoma. There was no pituitary stalk deviation. As he presented a clinical picture of true acromegaly, he underwent transsphenoidal surgical resection of mass. A pathological evaluation (Fig. 3) showed pituitary adenoma, with extensive expression of GH and ACTH, and rare expression of follicle-stimulating hormone (FSH) and PRL.

The genetics analyses of *AGPAT2* by polymerase chain reaction (PCR) and Sanger sequencing revealed absence of amplification of exons 3 and 4. In order to clarify this fact, deletions/duplications located in the *AGPAT2* gene (by quantitative PCR, using the PROBE Hs00085627-*AGPAT2* located at the transition intron 3/exon 3) demonstrated no amplification of the intron 3/exon 3 region for the *AGPAT2* gene, which pointed to an exon 3/exon 4 deletion of the *AGPAT2* gene in homozygosity, previously described [17, 18].

Our patient was prescribed a diet, exercise, lifestyle modification, metformin 700 mg twice a day, atorvastatin 20 mg, and levothyroxine 75 μg for diabetes, dyslipidemia, and CH, respectively. Three months after pituitary surgery, his IGF-1 level of 172 ng/mL (51–187 ng/mL; Table 4) and OGTT (Table 5) were normal, with no other hormonal abnormalities, namely other pituitary hypofunction.

**Table 4:** Hormonal parameters 3 months after pituitary surgery

Parameter	Result	Normal range
GH, ng/mL	2.34	< 3.0
IGF-1, ng/mL	172	51–187
IGFBP-3, μg/mL	5.7	3.2–6.9

*GH* somatotropin, *IGF-1* insulin-like growth factor-1, *IGFBP-3* insulin-like growth factor-binding protein 3

**Table 5:** Oral glucose tolerance test 3 months after pituitary surgery

OGTT	Glucose (mg/dL)	Insulin (μU/mL)	GH (ng/mL)
0 minutes	90	21.6	0.24
30 minutes	213	161.1	0.09
60 minutes	258	284.2	0.08
90 minutes	212	185.0	0.06
120 minutes	141	97.7	0.39

*GH* growth hormone, *OGGT* oral glucose tolerance test

## Discussion

This patient presented characteristics of generalized lipodystrophy, with lipoatrophy and associated metabolic consequences. As lipodystrophic phenotype had been present since birth, this led to the suspicion and diagnosis of CGL. Having identified *AGPAT2* deletion, BSCL type 1 was subsequently diagnosed. Although BSCL is generally recognized at birth, or shortly afterwards, as it is manifested by almost total body fat loss and prominent muscularity, which causes a serious and notable phenotype [10], in this particular case, our patient was only diagnosed as having the condition in adulthood. The delay in the diagnosis was probably due to the rarity of this clinical condition and the consequent lack of knowledge regarding it and the fact that there was an unusual absence of diabetes and dyslipidemia since puberty in this case. Our patient had many of the features of Berardinelli-Seip syndrome, such as: lipoatrophy affecting the trunk, limbs, and face; acromegaloid features; skeletal muscle hypertrophy; hypertriglyceridemia and insulin resistance; and hypertrophic cardiomyopathy and hepatomegaly secondary to hepatic steatosis [7]. The underlying etiology of cardiac abnormalities in lipodystrophy is still not clearly understood. Patients with CGL have severe insulin resistance, which may provide the context for the development of hypertrophic cardiomyopathy, given the effect of insulin on growth. Accumulation of triglycerides in the myocardium can also cause “lipotoxic cardiomyopathy”; however, there is no clear evidence that these patients have ectopic fat deposition in the myocardium [19]. In this particular patient, the presence of acromegaly may have contributed to both conditions (hypertrophic cardiomyopathy and hepatomegaly) [20].

The fact that our patient had normal TSH does not exclude CH, as 84% of patients with CH have normal TSH values [21] and the combination of a low FT4 and a non-markedly elevated TSH in patients with known pituitary disease is a diagnostic of CH, unless a patient is severely ill and is affected by non-thyroidal illness-induced changes in the thyroid hormone [22]. Both CGL and acromegaly could explain this finding. We could speculate that leptin, which has a significant impact on the synthesis and release of the thyrotropin-releasing hormone and is a metabolic signal that modulates hypothalamic–pituitary–thyroid axis activity in animals and humans, could explain the CH as both lipodystrophy and acromegaly are associated with low levels of leptin [23].

The mechanism underlying acromegaloid characteristics in lipodystrophies is not yet fully understood, although varied abnormalities in GH levels were reported. Uncontrolled diabetes leads to liver resistance to GH, with decreased hepatic IGF-I production. The lack of negative feedback effect of IGF-I on GH secretion causes GH hypersecretion, which partially explains the elevated GH level observed in some of these patients; however, this is not the case here, as HbA1c was controlled in our patient [24, 25]. Our patient presented acromegaloid characteristics, as well as acromegaly confirmed in histology [15]. The acromegaloid phenotype can be present in patients with CGL without acromegaly; however, in this case, the very marked acromegaloid features associated with a high degree of clinical suspicion of a true acromegaly and the duplications of IGF-1 levels (which are not usually common in CGL) led to the need to repeat the laboratorial evaluation. Even though Endocrine Society guidelines point out that with more sensitive new assays used for GH determination, a nadir of GH serum < 0.4 ng/mL after an orally administered glucose load could be used to exclude acromegaly, they suggest that it is sufficient to use the cut-off value < 1 ng/mL after an orally administered glucose load to exclude this disease. If we used the cut-off of 1 ng/mL, as suggested, our patient would have IGF-1/GH discrepancy [12]. New data even suggest a cut-off of < 0.3 ng/mL to exclude this disease [16]. When a discrepancy of GH/IGF-1 value is found during a diagnosis, we should really pursue close follow-up [26]. Normal GH suppression [12] with high IGF-1is not so uncommon nowadays. In a recent series report, 31% of 157 treatment-naive patients with acromegaly had elevated IGF-1 and normal 24 hours mean plasma GH levels. The percentage of GH/IGF-1 discrepancy has increased to 47% in more recent years [27]. Newer GH assays, which are based on monoclonal antibodies with higher specificity, tend to be more sensitive when compared with older polyclonal antisera. Following an assay-adjusted cut-off, we confirmed the diagnosis. Furthermore, the pituitary MRI and later histological examination confirmed the presence of an adenoma, with extensive GH expression. From this case we have learned that we should expect GH cut-offs of normality according to the assay we use, particularly when we use ultrasensitive assays [25]. More, we learned that, although this is a rare association, the presence of CGL should not exclude the possibility of simultaneous acromegaly.

## Conclusion

This case of CGL type 1 (*AGPAT2* deletions) with true acromegaly is the first to be reported in the literature. The diagnosis of acromegaly was challenging due to the GH/IGF-1 discrepancy, which was surpassed by the use of a cut-off which was adjusted to the assay. Although this is a rare association, the presence of CGL should not exclude the possibility of simultaneous acromegaly.

## Data Availability

The datasets generated during and/or analyzed during the current study are available from the corresponding author on reasonable request.

## Abbreviations

ACTH: Adrenocorticotropic hormone
*AGPAT2*: 1-acylglycerol-3-phosphate O-acyltransferase 2
BMI: Body mass index
BSCL: Berardinelli–Seip congenital lipodystrophy
CGL: Congenital generalized lipodystrophy
CH: Central hypothyroidism
FMR: Fat mass ratio
FSH: Follicle-stimulating hormone
FT4: Free thyroxine
GH: Growth hormone
HbA1c: Glycated hemoglobin
HDL: High-density lipoprotein
HOMA-IR: Homeostatic model assessment of insulin resistance
IGF-1: Insulin-like growth factor-1
LDL: Low-density lipoprotein
MRI: Magnetic resonance imaging
OGTT: Oral glucose tolerance test
PCR: Polymerase chain reaction
PRL: Prolactin
TSH: Thyroid-stimulating hormone
